# CCT8 recovers WTp53-suppressed cell cycle evolution and EMT to promote colorectal cancer progression

**DOI:** 10.1038/s41389-021-00374-3

**Published:** 2021-12-03

**Authors:** Qing Liao, Yun Ren, Yuyi Yang, Xiaohui Zhu, Yunfei Zhi, Yujie Zhang, Yi Chen, Yanqing Ding, Liang Zhao

**Affiliations:** 1 Department of Pathology, Nanfang Hospital, Southern Medical University, Guangzhou, Guangdong Province People’s Republic of China; 2 Department of Pathology & Guangdong Province Key Laboratory of Molecular Tumor Pathology, School of Basic Medical Sciences, Southern Medical University, Guangzhou, Guangdong province People’s Republic of China; 3 Department of Pathology, Affiliated Tumor Hospital of Guangzhou, Medical University, Guangzhou, China; 4 The First School of Clinical Medicine, Southern Medical University, Guangzhou, Guangdong Province People’s Republic of China

**Keywords:** Cell migration, Colorectal cancer

## Abstract

LIM and SH3 protein 1 (LASP1) is a metastasis-related protein reported to enhance tumor progression in colorectal cancer (CRC). However, the underlying mechanism is still elusive. The chaperonin protein containing TCP1 (CCT) is a cellular molecular chaperone complex, which is necessary for the correct folding of many proteins. It contains eight subunits, CCT1-8. CCT8 is overexpressed in many cancers, however, studies on CCT8 are limited and its role on CRC development and progression remains elusive. In this study, we confirmed that CCT8 and LASP1 can interact with each other and express positively in CRC cells. CCT8 could recover the ability of LASP1 to promote the invasion of CRC; CCT8 could significantly promote the proliferation, invasion, and metastasis of colorectal cells in vivo and in vitro. Mechanically, CCT8 inhibited the entry of WTp53 into the nucleus, and there was a negative correlation between the expression of CCT8 and the nuclear expression of WTp53 in clinical colorectal tissues. CCT8 promoted the cell cycle evolution and EMT progression of CRC by inhibiting the entry of WTp53 into the nucleus. Clinically, CCT8 was highly expressed in CRC. More importantly, the overall survival of CRC patients with high expression of CCT8 was worse than that of patients with low expression of CCT8. These findings indicate that as LASP1-modulated proteins, CCT8 plays a key role in promoting the progression of colorectal cancer, which provides a potential target for clinical intervention in patients with colorectal cancer.

## Introduction

Colorectal cancer (CRC) is one of the most common malignant tumors and the third leading cause of cancer-related death worldwide [1]. Metastasis is widely believed to be responsible for the high mortality and poor prognosis. The occurrence and development of CRC is a multi-step process driven by multiple genes and factors. Therefore, identifying the new proteins involved in the development of CRC and exploring its mechanism will help to reveal the molecular mechanism of the occurrence and development of CRC, providing a potential opportunity for the treatment of this fatal disease.

The cytoplasmic chaperonin containing TCP1 complex (CCT), also known as the TCP1 ring complex (TRiC) consists of two identical stacked rings, each containing eight different proteins (CCT1-CCT8). They are products encoded by a single gene, and the molecular weight of each subunit is about 60 kDa. The main substrates of CCT may be cytoskeletal proteins, such as actin and tubulin [2, 3]. CCT plays an important role in maintaining cellular homoeostasis by assisting the folding of many proteins [4]. CCT8 is a subunit of the CCT complex, which contains gene sequences common to all other subunits of CCT related to ATP enzyme activity [5]. Many studies have shown that CCT, especially subunit 8 (CCT8), plays an important role in the tumor progression of B cell non-Hodgkin’s lymphoma [6] and glioma [7]. In addition, studies have also shown that CCT8 is overexpressed in colon cancer and hepatocellular carcinoma [8]. However, the mechanism of CCT8 in the occurrence and development of colorectal cancer is not clear.

LIM and SH3 protein 1 (LASP1) were originally identified from the cDNA library of breast cancer patients with axillary lymph node metastasis [9]. Human LASP1 gene encodes a protein of 261 amino acids, which contains an N-terminal LIM domain and two actin binding domains. Thus, most biological functions of LASP1 might be accomplished by protein–protein interactions via the two functional domains [10]. Abnormal LASP1 expression has also been detected in several other cancer types, including prostate, pancreatic, ovarian, liver and nasopharyngeal cancers [11–15]. LASP1 has been identified as a protein associated with CRC metastasis in our previous studies, which can promote the progression of CRC and lead to poor clinical outcomes [16]. The above-mentioned CCT8 is the protein regulated by LASP1 selected by us through the two-dimensional differential gel electrophoresis (2-D DIGE) in CRC cell lines.

Currently, the roles of LASP1-CCT8 axis in the progression of CRC and the specific molecular mechanism of CCT8 in the occurrence and development of CRC have not been systematically studied. Therefore, this study aimed to determine the function of LASP1-CCT8 axis in aggressive CRC. We have determined the interaction between CCT8 and LASP1, CCT8 could antagonize the cell cycle arrest and EMT transcriptional inhibition of WTp53 by inhibiting the entry of WTp53 into the nucleus, thus promote the proliferation, invasion and metastasis of CRC.

## Materials and methods

### Cell culture and treatment

CRC cell lines including RKO, HCT116, SW480, SW620, HCT15, DLD1, HT29 and Caco2 were obtained from Cell Bank of the Chinese Academy of Sciences (Shanghai, China), and maintained as previously described [16]. All the cells were cultured in RPMI 1640 (Hyclone; Logan, Utah, USA) with 10% fetal bovine serum (FBS) (Gibco-BRL, Invitrogen; Paisley, UK) at 37 °C with a humidity of 5% CO_2_. Plasmids including pcDNA3-LASP1, and pcDNA3-CCT8 were constructed in our lab. All siRNA oligos including LASP1 and CCT8 specific siRNAs were purchased from GenePharma (Shanghai, China). The siRNA oligos used are shown in Supplementary Table S1. CRC cells at exponential growth phase were plated into 6-well plates for 24 h at a density of 0.5 × 10^5^ cells/mL, and transfected with 1 mg of siRNA or 4 µg cDNA in reduced serum medium (OPTI-MEM-I; Invitrogen) according to the manufacturer’s protocol.

### Clinical samples

A total of 145 tumor tissue samples from patients with CRC, and 37 matched normal tissue samples were obtained from the tumor tissue bank of Southern Hospital. Each patient was diagnosed with primary CRC. All the patients did not receive radiotherapy and chemotherapy before operation and there was no history of other malignant tumors. Each patient’s clinical data and follow-up data were completed. The clinicopathological information such as tumor clinical stage and TNM stage was extracted from the case analysis. All the samples were randomly selected, except for the integrity of patient follow-up data may lead to the emergence of potential self-selection bias, other factors will not lead to self-selection bias. The study was approved by the Ethics Committee of Southern Medical University and all aspects of the study are in line with the Declaration of Helsinki. No informed consent was required because data were analyzed anonymously.

### TMA construction

Screening hematoxylin- and eosin-stained slides for optimal tumor tissue and tumor adjacent tissue up to 2 cm from the tumor, TMA slides were constructed. Two cores were taken from each formalin-fixed, paraffin-embedded tumor and matched normal colon tissue specimen as well as at least one lymph node metastasis core using punch cores that measured 2 mm in greatest dimension from the nonnecrotic areas of tumor, lymph node metastasis, and matched normal colon tissue specimens. These specimens were included in the TMA.

### Immunohistochemistry (IHC) and assessment of protein expression

Immunohistochemistry was performed, as previously described [17], to investigate the expression of CCT8 and its related proteins in human CRC tissues. The sections were incubated overnight using primary antibodies against CCT8 (1:500; Santa Cruz, California, USA), LASP1(1:100; Proteintech, Chicago, IL), p53 (1:4000; Proteintech, Chicago, IL) and MTp53 (1:1000; Gene Tech, Shanghai, China) at 4 °C. Mayer’s haematoxylin was used for nuclear counterstaining. The immunohistochemical staining was scored semi-quantitatively accompanied by the staining intensity of positive tumor cells. Signal intensity was scored as 0(negative), 1 (weak), 2 (moderate) or 3 (strong). The expression ratio was scored as 1 (0–25%), 2 (26–50%), 3 (51–75%) or 4 (76–100%). The immunostaining score was defined as 0 (scored 0–3), 1 (scored 4–6), 2 (scored 6–9) or 3 (scored 9–12), according to the multiplication of signal intensity and expression ratio scores. Among these grades, 0–1 was defined as low expression, and 2–3 was defined as high expression. In this study, these slides were reviewed by two or three blinded pathologists. The discrepancies (<5%) were resolved by simultaneous re-evaluation. The Spearman correlation analysis was used to determine the significance of correlation.

### Assessment of p53 Expression

To estimate p53 expression, we examined specimens for p53 protein by immunohistochemistry. P53(1:4000; Proteintech, Chicago, IL) were used to detect total p53 protein, including WTp53 and MTp53. Slides were examined for MTp53 reactivity using the MTp53(1:1000; Gene Tech, Shanghai, China) and estimated semi-quantitatively. Cases with ≥ 50% moderate/strong nuclear staining of the tumor cells were considered MTp53 cases(MTp53 positive) [18], as recommended for improved specificity [19]. The WTp53 cases (MTp53 negativity) was defined as either absent/weak staining or ≤50% of tumor cells with moderate/strong MTp53 staining but with moderate/strong total p53 staining. This was based on many previous studies, which showed that MTp53 positive nuclear staining correlates well with TP53 gene missense mutation that involve more than 80% of p53 alterations [20]. Although the concordance between MTp53 overexpression and mutations is not perfect, the immunohistochemical assay is a rapid and cost-effective evaluation. In comparative studies, MTp53 overexpression is a good approximation of the real mutation rate, with approximately 90% concordance between immunostaining and gene analysis and 91% specificity [21, 22].

### Animals

Twenty female BALB/c nude mice at 3–5 weeks of age were purchased from Southern Medical University Experimental Animal Centre. All animal experiments were approved by the Committee for Animal Care and Use Committee of Southern Medical University in accordance with animal ethical treatment guidelines. All animal experiments have received permits for ethical and humane treatment issued by the Department of Science and Technology of Guangdong Province. The experimental mice were fed with an autoclaved laboratory rodent diet and maintained in a barrier facility in racks filtered with high-efficiency particulate air filter. For the construction of subcutaneous colon cancer xenograft model, 5 × 10^6^ cells were injected into the mammary fat pad of each nude mouse. After 4 weeks, mice were sacrificed by cervical dislocation and the xenograft tumors were quickly harvested for histological study. For the construction of metastasis model, 1 × 10^7^ cells (100 μl) were injected into the cecum of each nude mice. After 4 weeks, the nude mice were dissected; liver tissues were collected. The tumor volume was calculated according to the formula: Volume (mm^3^) = width^2^ (mm^2^) × length (mm)/2. The liver tissue was stained with hematoxylin- and eosin, the nodules metastasized from cecal tumor to liver tissue were counted under 10× low power microscope, the number of tumor nodules were recorded and analyzed. For the in vivo experiments related to animals, the number of biological replicates is 5. Animals were allocated to control experimental groups using a blinding and randomization method.

### Western blot analysis

Protein expression was assessed by immunoblot analysis of cell lysates (20–60 µg) in RIPA buffer in the presence of mouse antibodies to CCT8, E-cadherin, N-cadherin, vimentin, slug (1:1000; Santa Cruz, California, USA); rabbit antibodies against LASP1, p53, ubiquitin, P21, P27, Cycline D1, CDK4, CDK6, myc, bax, snail, twist, β-catenin (1:1000; Proteintech, Chicago, IL); mouse antibodies to β-actin, GAPDH (1:1000; ZSGV-BIO, Beijing, China). Bands were quantified by the densitometry function of the Quantity One software. β-actin and GAPDH were used as an endogenous control. All bands were normalized to internal controls, and fold changes were calculated through relative quantification.

### RNA isolation, reverse transcription and real-time quantitative PCR

Total RNA was extracted using Trizol reagent (Invitrogen). To quantitate the expression of target genes, total RNA was polyadenylated and underwent reverse transcription. Real-time quantitative PCR (qPCR) was carried out using a SYBR® Premix Ex TaqTM II (TaKaRa, Dalian, China) on an ABI 7500HT system. All samples were normalized to internal controls, and fold changes were calculated through relative quantification (2^−ΔΔCT^). Real-time PCR for target genes was performed as previously described [23]. The primers used are shown in Supplementary Table S2.

### Data analysis

Data were analyzed using SPSS statistical software version 19.0 (SPSS; Chicago, USA). All values are expressed as the mean ± standard deviation. For the in vitro experiments, two different cell lines were included and the conclusion was obtained from three individual experiments, but only the representative figure is displayed. The statistical analysis performed for the in vitro studies was unpaired t-test. Student’s t test and one-way ANOVA were carried out for RT-PCR. The significance of the correlation between the expression of CCT8 and histopathological factors was determined using Pearson’s chi-squared (χ^2^) test. The correlation between CCT8, LASP1 and p53 in paraffin-embedded clinical tumor tissues were performed using Spearman’s correlation analysis. Statistical significance was established at *P* < 0.05.

## Results

### LASP1 positively regulates CCT8 expression by protein interactions

**Fig. 1:**
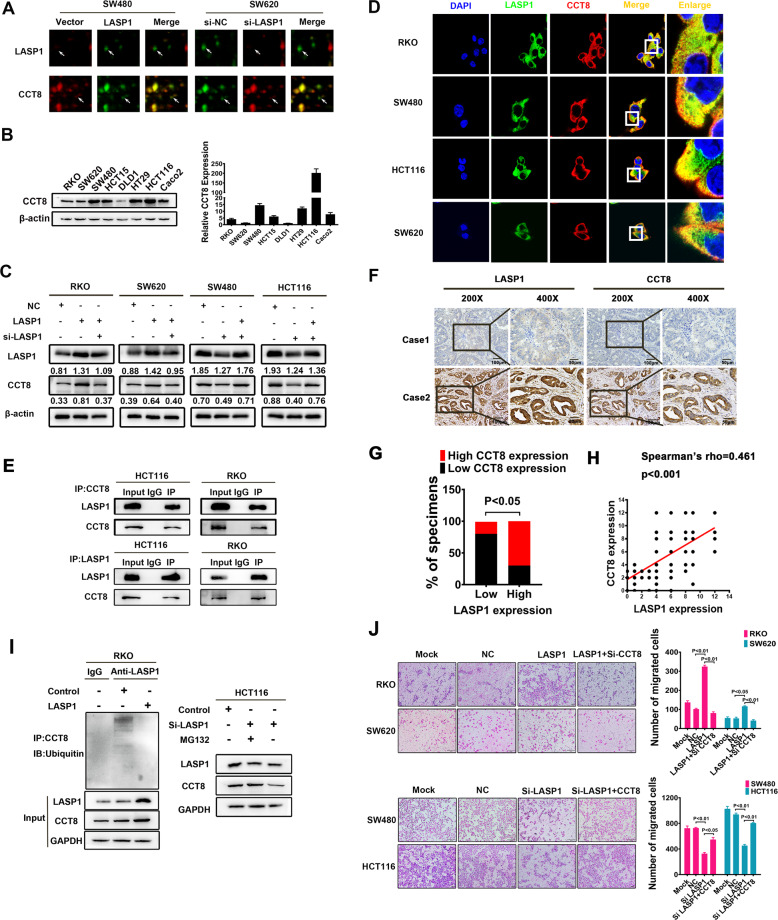
LASP1 positively regulates CCT8 expression by protein interactions. **A** The differentially expressed proteins in SW480/LASP1 cells or SW620/si-LASP1 and control cells were screened by 2-D DIGE. The enlarged images of two differentially expressed protein spots in DIGE analysis were shown. The protein spots were indicated by white arrows. **B** Western Blot analysis was performed to detect the expression of CCT8 in CRC cell lines(left). Real-time qPCR was used to detect the express of CCT8 in CRC cells at transcriptional level(right). **C** Western Blot was performed to detect the expression of CCT8 and LASP1 protein in indicated cells. **D** The immunofluorescence staining was performed to assess the subcellular localization of CCT8 and LASP1 in indicated cells (original magnification ×2400). The box areas highlighted the co-localization between CCT8 and LASP1. **E** The Co-IP was performed to analyze the endogenous interaction between CCT8 and LASP1 in CRC cells. Cells were lysed and purified by anti-CCT8 or anti-LASP1 affinity gel; protein pellets were analyzed by Western Blot with anti-LASP1 and anti-CCT8 antibodies. **F** Paraffin-embedded CRC sections were stained with anti-CCT8 or anti-LASP1 antibodies. Visualization of two representative cases was shown. **G** Graphical illustration of statistical LASP1 and CCT8 distribution in CRC tissues. The high expression of CCT8 is more frequently accompanied with high expression of LASP1 (*p* < 0.05). **H** Scatter plot display of the correlation between LASP1 and CCT8. **I** LASP1 suppressed the ubiquitination of CCT8 and the following degradation of CCT8. Left panel: Cells were lysed and purified by anti-CCT8 affinity gel and protein pellets were analyzed by Western Blot with anti-ubiquitin. Right panel: MG132 recovered the si-LASP1 increased degradation of CCT8. **J** Representative figures and data of Transwell assay in indicated cells. Bars of the right panel represented the number of invaded cells.

In order to explore downstream molecules regulated by LASP1, we performed a proteomics strategy based on 2-D difference gel electrophoresis (2-D DIGE) (Fig. S1). And one of the candidates LASP1-modulated proteins was identified as TCP1 complex subunit 8 (CCT8), which was positively correlated with LASP1 expression (Fig. 1A). Western Blot and RT-PCR were performed to detect the expression of CCT8. The results indicated that: CCT8 was expressed in all CRC cell lines, with relatively low expression in RKO, SW620, and DLD1 cell lines and relatively high expression in SW480, HCT15, HT29, HCT116, and Caco2 cell lines (Fig. 1B). Western blot analysis detected that CCT8 expression was enhanced in LASP1-overexpressed RKO and SW620 cells, while weakened in LASP1-silenced SW480 and HCT116 cells, which was consistent with proteomic analysis. And the change of CCT8 can be restored by the interference or overexpression of LASP1 (Fig. 1C). More interestingly, we observed co-localization of membrane dye Dio, LASP1 and CCT8 in RKO, SW480, HCT116, and SW620 cells, immunofluorescence staining showed that the co-localization of LASP1 and CCT8 were mainly in cell cytoplasm (Fig. 1D and Fig. S2). The protein interaction was also verified by Co-IP assay in protein extraction from HCT116 and RKO cells (Fig. 1E). However, this protein interaction did not affect the expression of LASP1 protein (Fig. S3). To investigate the relationship between LASP1 and CCT8 in clinical tissues samples, immunohistochemical assay was performed and showed that CCT8 and LASP1 expressions were concordant in most CRC cases (Fig. 1F, G). CCT8 was positively correlated with the LASP1 expression, obviously (Fig. 1H; Spearman’s rho = 0.461, *P* < 0.001). To further explore the regulatory mechanism of LASP1, we examined the effects of LASP1 on transcriptional activation and protein stability of CCT8. The results showed that LASP1 did not affect CCT8 mRNA expression (Fig. S4), but inhibited ubiquitin-mediated degradation of CCT8 (Fig. 1I). Our previous studies showed that overexpression of LASP1 promoted the invasion ability of CRC cells [16]. To explore the pivotal role of LASP1-CCT8 axis in the LASP1-mediated cell aggressive phenotype, we conducted a rescued experiment to detect the invasion ability of CRC cells. The results showed that loss of CCT8 attenuated cell migration and reversed LASP1-induced migration of CRC cells, while restoring expression of CCT8 recovered the invasion ability of CRC cells (Fig. 1J).

### CCT8 promotes CRC cell migration in vitro and metastasis in vivo

**Fig. 2:**
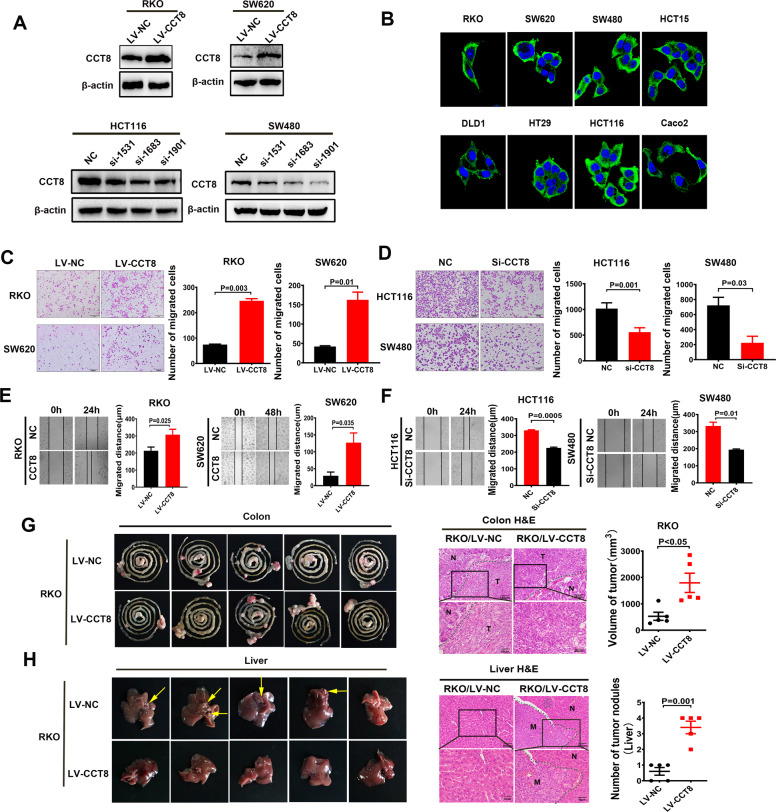
CCT8 promotes CRC cell migration in vitro and metastasis in vivo. **A** CCT8 overexpressed lentivirus and three CCT8 siRNA were successfully constructed to study the effects of CCT8 in CRC cells. **B** The subcellular localization of CCT8 in CRC cells lines was assessed by immuno-fluorescence. **C**, **D** Representative figures of Transwell assays indicate the stimulatory role of CCT8 on CRC migration. Bars on the right panel represent the number of invaded cells (*n* = 3, *P* < 0.05). **E**, **F** Representative figures of the wound-sealing assays indicate that CCT8 could stimulate CRC cell migration. Bars on the right represent the migration distance of wound-healing assay (*n* = 3, *P* < 0.05). **G**, **H** Caeca orthotopic transplantation assay indicates the stimulatory role of CCT8 on hepatic metastasis (*n* = 5). The representative colon and liver samples are displayed and the HE staining of the representative tumors was placed on the middle panel, showing the boundaries between tumor and normal tissues. Bars of the right panel represented the volume of tumor in situ and number of metastatic nodules in liver respectively.

To study the biological effects of CCT8, we constructed CRC cell lines with transient knockdown, transient overexpression and stable overexpression of CCT8. We successfully constructed the CCT8 overexpressing lentiviral vector, and synthesized two CCT8 siRNAs to explore their effects on CRC cells. Western blot results verified that all CRC cell lines were successfully constructed (Fig. 2A). Immunofluorescence staining showed that CCT8 was mainly expressed in the cytoplasm of CRC cells (Fig. 2B). Transwell and wound-healing assays showed that exogenous CCT8 significantly increased migration and motility capacity of CRC cells in vitro (Fig. 2C, E). In contrast, si-CCT8 significantly reduced the aggressive capacity of CRC cells (Fig. 2D, F). RKO cells with stable and overexpression of CCT8 were injected into the caecum of nude mice to observe its effect on liver metastasis. Compared with control group, more and larger tumor nodules were formed in situ and in the liver in the RKO/LV-NC group (Fig. 2G, H). The data in vivo further supports CCT8 as a metastatic enhancer.

### CCT8 promoted the proliferation of CRC cells in vivo and in vitro

**Fig. 3:**
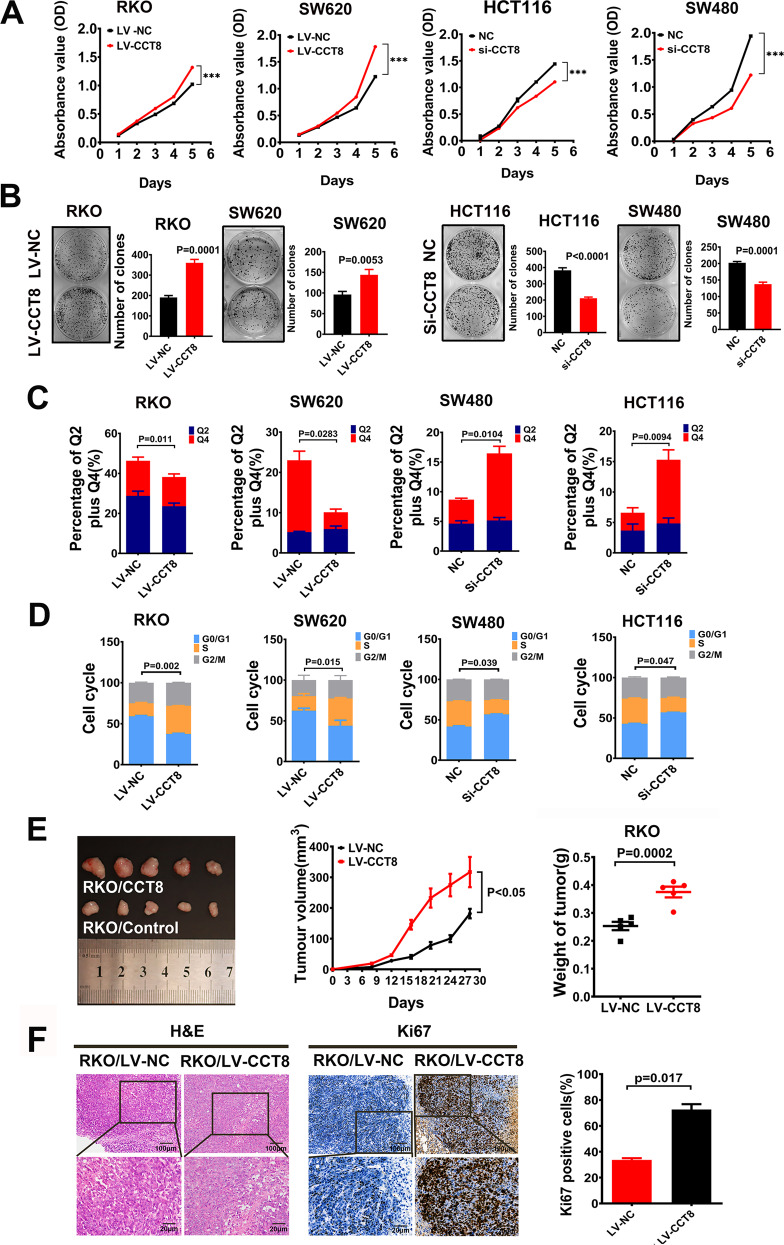
CCT8 promoted the proliferation of CRC cells in vivo and in vitro. **A** The CCK8 assay was performed to study the effects of CCT8 on CRC cell proliferation in loss- and gain-of-function analysis. **B** Representative figures and data of colony formation assay in indicated cells. Bars of the right panel represented the number of formed cells. **C** Bars represent apoptosis in vitro after treatment of fluorouracil for 12 h. **D** Bars represent the cell cycle analysis in CCT8 overexpressed and silenced CRC cell lines. **E** Resected xenograft tumors were injected with RKO/LV-CCT8 and RK0/LV-NC. The tumor growth curve over time and the final tumor weight in the scatter plot graph. **F** Representative figures of HE staining of subcutaneous tumor were shown. Proliferative ability was measured by the Ki-67 proliferative labeling index. Bars of the right panel represent the Ki-67 index of subcutaneous tumor.

To further clarify the role of CCT8 in the progression of CRC, the effect of CCT8 on the proliferation of CRC cells was detected by CCK-8 assay and clone formation assay. The results showed that the proliferation of CRC cells was significantly enhanced in the group with overexpression of CCT8 (Fig. 3A, B). After the treatment of fluorouracil in CRC cell lines for 12h, the effect of CCT8 on the apoptosis of CRC cell lines was detected by flow cytometry. The results showed that overexpression of CCT8 could significantly reduce the proportion of apoptosis of CRC cell lines, while depletion with CCT8 could significantly increase the proportion of apoptosis of CRC cell lines (Fig. 3C, Fig. S5). Cell cycle analysis showed that CCT8 could promote CRC cells from G0/G1 phase to S phase, thus promoting cell proliferation. On the contrary, knocking down CCT8 tended to keep cells staying in G0/G1 phase (Fig. 3D and Fig. S6). Then, a subcutaneous tumor model was performed to assess the ability of tumor genesis and growth. RKO/LV-CCT8 and RKO/LV-NC were injected subcutaneously into BALB/c nude mice, respectively. The results showed that the subcutaneous tumors in the RKO/LV-CCT8 group grew obviously faster (Fig. 3E) and showed a higher percentage of positive Ki-67 tumor cells compared with those in the RKO/LV-NC group (Fig. 3F).

### CCT8 inhibits the entry of WTp53 into the nucleus and antagonizes its anti-tumor effect

**Fig. 4:**
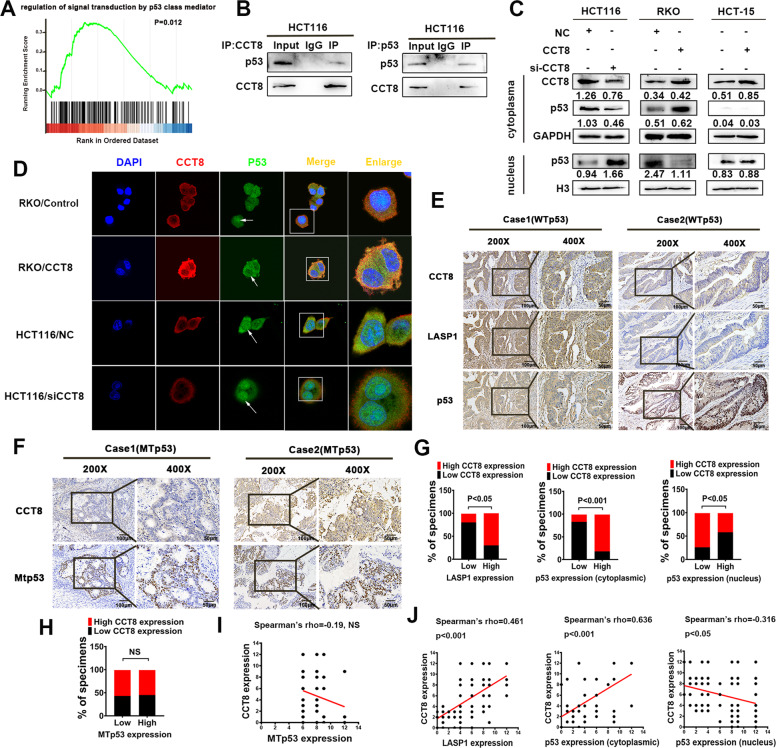
CCT8 inhibits the entry of WTp53 into the nucleus and antagonizes its anti-tumor effect. **A** GSEA demonstrated enrichment of p53 signal pathway in high CCT8 expression CRC group. (*p* = 0.012). **B** The Co-IP was performed to analyze the endogenous interaction between CCT8 and p53 in CRC cells. Cells were lysed and purified by anti-CCT8 or anti-p53 affinity gel; protein pellets were analyzed by Western Blot with anti-p53 and anti-CCT8 antibodies. **C** Effect of CCT8 on the expression of p53 were detected by Western Blotting analysis (a representative set of data was shown, *n* = 3). **D** Immunofluorescence staining showed that CCT8 could inhibit the entry of p53 into the nucleus. The arrow shows nuclear p53. The box areas highlighted the co-localization between CCT8 and p53. **E** Paraffin-embedded CRC sections were stained with anti-CCT8, anti-LASP1 or anti-p53 antibodies. Visualization of two representative cases was shown. **F** Paraffin-embedded CRC sections were stained with anti-MTp53 antibodies. Visualization of two representative cases was shown. **G** Graphical illustration of statistical LASP1, CCT8 and p53 distribution in CRC tissues. The high expression of p53 in cytoplasmic is more frequently accompanied with high CCT8 in WTp53 cases (*p* < 0.05). **H** Graphical illustration of statistical CCT8 and MTp53 distribution in CRC tissues. The expression CCT8 was in-dependent of MTp53 expression (NS). **I** Scatter plot display of the correlation between CCT8 and MTp53(NS). **J** Scatter plot display of the correlation between CCT8 and p53.

In order to explore the CCT8-related signal pathway, we analyzed the CRC-related gene expression data in the public database TCGA (*n* = 478) (https://portal.gdc.cancer.gov), and found that p53 signal pathway (GO:1901796) was enriched in CCT8 high expression group (gene set enrichment analysis, GSEA) (*p* value = 0.012; *q* value = 0.025) (Fig. 4A). To verify the relationship between CCT8 and p53, the cytoplasmic nuclear proteins were separated in HCT116 cell line. The interaction between CCT8 and p53 in the cytoplasm was detected by Co-IP assay (Fig. 4B). At the same time, we examined the effect of CCT8 on p53. The results of WTp53 cell line HCT116 and RKO showed that knocking down CCT8 decreased the expression of p53 in cytoplasm and increased the expression of p53 in nucleus, while overexpression of CCT8 could block p53 in the cytoplasm and inhibit its entry into the nucleus; in p53 mutant cell line HCT15, p53 was mainly expressed in nucleus, but not in cytoplasm, and overexpression of CCT8 did not regulate the expression of p53 (Fig. 4C). Immuno-fluorescence assay results of WTp53 cell lines further confirmed that overexpression of CCT8 inhibited the entry of p53 into the nucleus, while knocking down CCT8 promoted the entry of p53 into the nucleus (Fig. 4D). In addition, we detected the expression of CCT8 and p53 in clinical tissues. The immunohistochemical results showed that some expression pattern of p53 were localized in cytoplasmic and regularly accompanied with high expression levels of CCT8, while other expression pattern of p53 were localized in nuclei and more inclined to accompany with low expression levels of CCT8 (Fig. 4E & G). Correlation analysis shows that the expression of CCT8 was positively correlated with the cytoplasmic expression of p53 (Fig. 4J, Middle; Spearman’s rho = 0.636, *P* < 0.001) and negatively correlated with the nuclear expression of p53 in WTp53 cases (Fig. 4J, Right; Spearman’s rho = −0.316, *P* < 0.05). However, there was no significant correlation between the expression of CCT8 and the nuclear expression of MTp53 (Fig. 4F, H and I; R = −0.019, NS).

### CCT8 antagonizes WTp53 mediated cell cycle arrest and EMT transcriptional inhibition

**Fig. 5:**
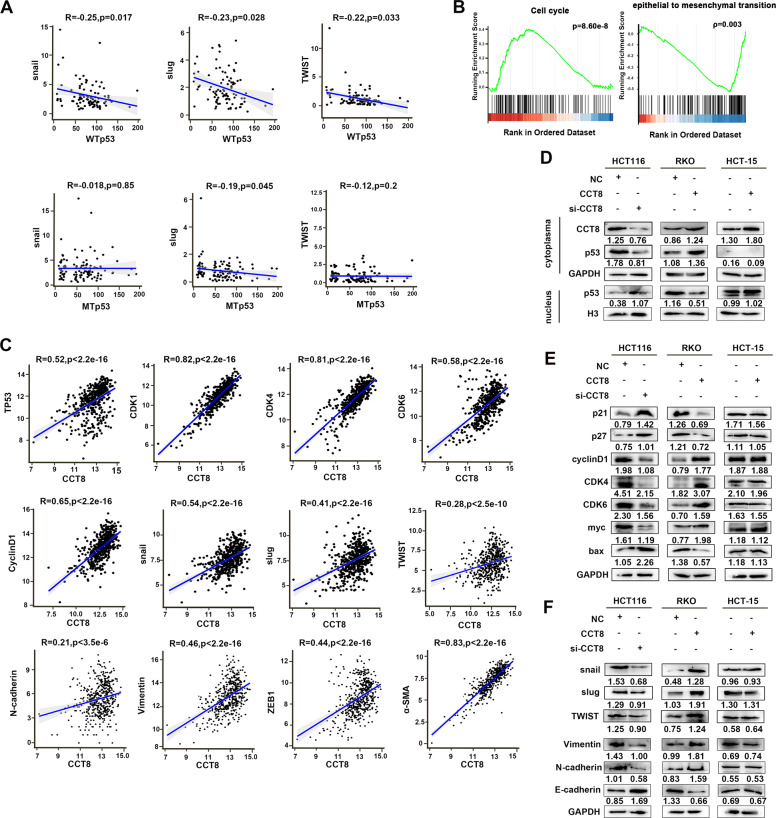
CCT8 antagonizes WT p53 induced cell cycle arrest and EMT transcriptional inhibition. **A** The correlations between EMT transcription factors (snail, slug, and twist) and WTp53/MTp53. **B** GSEA demonstrated cell cycle and EMT transformation were closely related to the expression of CCT8. **C** The correlations between CCT8 and p53, cell cycle associated gene (CDK1, CDK4, CDK6, and CyclinD1) and EMT related markers (snail, slug, twist, N-cadherin, Vimentin, ZEB1 and α-SMA). **D** Effect of CCT8 on the expression of p53 were detected by Western Blotting analysis (a representative set of data was shown, *n* = 3). **E**, **F** Western Blot analysis was performed to detect the protein expression of cell cycle pathway and epithelial-mesenchymal transition (EMT).

Using the gene expression data downloaded from TCGA, we found that WTp53 was negatively correlated with EMT transcription factors such as snail, slug, and twist, but not or weakly correlated with MTp53 (Fig. 5A). It is worth noting that gene enrichment analysis (gene set enrichment analysis, GSEA) using CRC-related gene expression data (478 cases) in the public database TCGA (https://portal.gdc.cancer.gov/) also found that cell cycle (hsa04110) and EMT transformation (GO:0001837) were closely related to the expression of CCT8 (*p* value = 8.60e-8, *q* value = 9.33e-7; *p* value = 0.003, *q* value = 0.005) (Fig. 5B). Further correlation analysis using TCGA database showed that p53, cell cycle associated gene and EMT related markers were positively correlated with the expression of CCT8 in CRC (*p* < 0.05) (Fig. 5C). Nuclear cytoplasmic protein separation experiment and Western blot analysis demonstrated that silencing CCT8 increased the expression of p53 in nucleus, up-regulated the expression of cell cycle inhibitory protein (p21, P27) and apoptosis protein Bax, and decreased the expression of cell cycle related molecules (CDK4, CDK6, cyclinD1, myc). On the contrary, after overexpression of CCT8, the expression of cell cycle related molecules was restored, and the expression of apoptosis protein Bax was decreased. Interestingly, in p53 mutant HCT-15 cell, p53 was mainly expressed in the nucleus, and CCT8 did not regulate the expression of cell cycle related molecules and apoptotic protein Bax (Fig. 5D, E). We also detected EMT-related proteins, immunoblot assay resulting in a gain of EMT transcription factors (snail, slug, and twist) and mesenchymal markers (vimentin, N-cadherin), and a loss of epithelial marker (E-cadherin) in CCT8 overexpressed RKO cells; On the contrary, si-CCT8 inhibited the EMT process in HCT116 cells; However, CCT8 did not affect the expression of EMT-related molecules in p53 mutant HCT-15 cell (Fig. 5F). These results suggested that CCT8 may enhance the proliferation and migration of CRC cells by promoting cell cycle evolution and EMT process.

### High expression of CCT8 is associated with the progression and poor prognosis of colorectal cancer

**Fig. 6:**
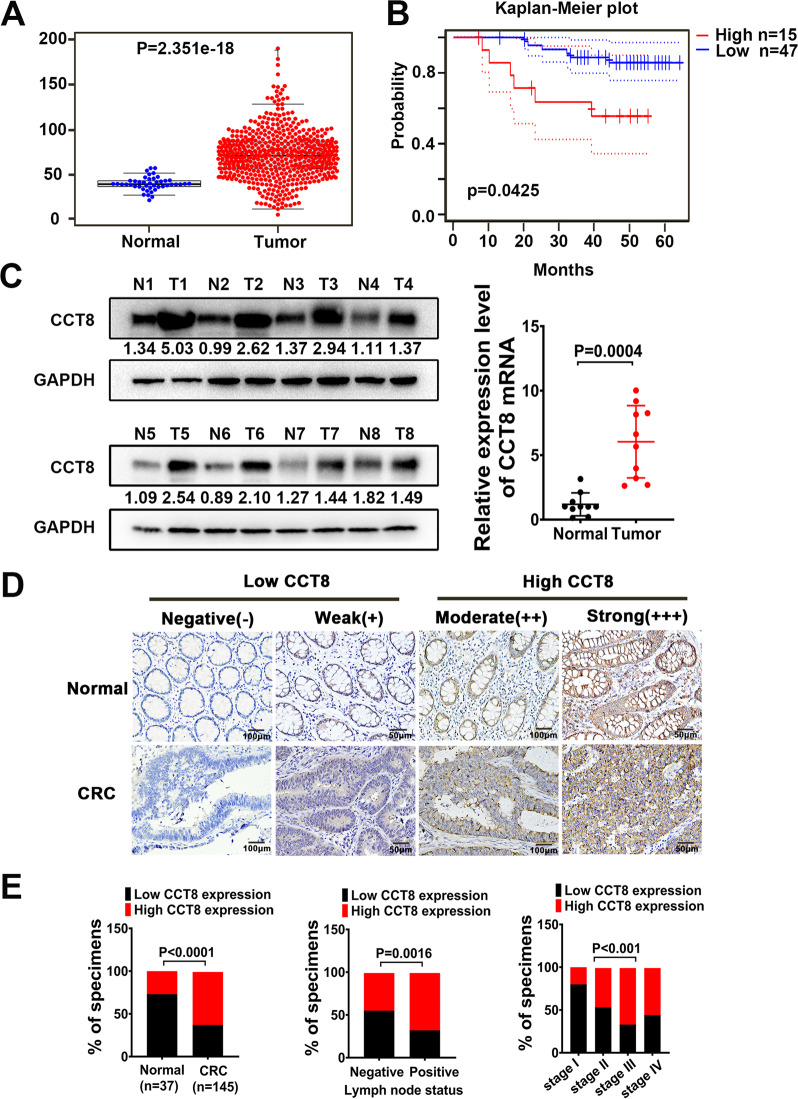
High expression of CCT8 prompts the progression and poor prognosis of colorectal cancer. **A** CCT8 gene expression from TCGA CRC database. (*p* = 2.351e-18). **B** Kaplan-Meier survival curves and univariate analyses(log-rank) of CRC samples obtained from PrognoScan database with distinct expression level of CCT8.(*p* = 0.0425). **C** Western Blot analysis was performed to detect CCT8 protein expression in CRC tissues (T) and adjacent non-tumor tissues (N). RT-PCR was performed to detect the CCT8 mRNA expression in CRC tissues and adjacent non-tumor tissues. **D** The expression of CCT8 in CRC tissues and adjacent nontumor tissues. **E** Graphical illustration of statistical CCT8 distribution in CRC tissues. CCT8 was significantly higher in CRC than in adjacent nontumorous tissue. The high expression of CCT8 was more frequently found in CRC with lymph node metastasis than in CRC without lymph node metastasis (N0). The high expression of CCT8 was more frequently found in advanced CRC (Stage I < Stage II < Stage III).

In order to analyze the difference of CCT8 expression between CRC and normal tissues, the expression of CCT8 was extracted by using the expression data of colorectal oncogene in public database TCGA (https://portal.gdc.cancer.gov/), and the difference scatter map was drawn. The results showed that the expression of CCT8 was significantly higher in CRC than that in normal tissues (*P* < 0.05) (Fig. 6A). Furthermore, Western blot and RT-PCR were used to detect fresh colorectal tumor tissue samples and paired normal tissue samples. The results showed that the expression of CCT8 in fresh CRC tissues was significantly higher than that in normal tissues (Fig. 6C). In order to evaluate the clinical significance of CCT8, immunohistochemical (IHC) detection was performed in 37 archived paraffin-embedded colorectal mucosa and 145 CRC tissues. Semi-quantitative immunohistochemical staining was performed based on the staining intensity of positive tumor cells. Negative (−) and weak (+) samples were rated as low CCT8, while medium (++) and strong (+++) samples were rated as high CCT8 (Fig. 6D, Fig. S7). CCT8 was overexpressed in 27% (10/37) normal colorectal samples, and a higher rate was observed in 62.8% (91/135) CRC samples (*P* < 0.001). The overexpression of CCT8 is closely related to lymph node metastasis (*P* = 0.0016) and clinical stage (*P* < 0.001) of CRC (Fig. 6E and Table 1). At the same time, we used the GSE12945 data set (GSE1294562) (http://dna00.bio.kyutech.ac.jp/PrognoScan/index.html) in PrognoScan database for Kaplan–Meier analysis. The results showed that the overall survival rate of colorectal cancer patients with high expression of CCT8 was significantly lower than that of patients with low expression of CCT8 (*P* = 0.0425) (Fig. 6B).

**Table 1:** Correlation of different parameters with CCT8 and LASP1 expression.

Characteristic	CCT8			LASP1		
	Low (%)	High (%)	*P* value	Low (%)	High (%)	*P* value
Normal	27 (73)	10 (27)	0.000	11 (29.7)	26 (70.3)	0.861
Tumour	54 (37.2)	91 (62.8)		41 (28.3)	104 (71.7)	
Gender						
Male	56 (45.5)	67 (54.5)	0.688	32 (26)	91 (74)	0.271
Female	25 (42.4)	34 (57.6)		20 (33.9)	39 (66.1)	
Age						
<50	16 (39)	25 (61)	0.422	9 (22)	32 (78)	0.286
≥50	65 (46.1)	76 (53.9)		43 (30.5)	98 (69.5)	
Site						
Colon	46 (42.2)	63 (57.8)	0.445	29 (26.6)	80 (73.4)	0.473
Rectal	35 (47.9)	38 (52.1)		23 (31.5)	50 (68.5)	
Size (cm in diameter)						
<5	49 (43.7)	63 (56.3)	0.795	30 (26.8)	82 (73.2)	0.5
≥5	32 (45.7)	38 (54.3)		22 (31.4)	48 (68.6)	
T classification						
T1 + T2	3 (18.8)	13 (81.3)	0.068	9 (56.3)	7 (43.8)	0.01
T3 + T4	70 (42.2)	96 (57.8)		43 (25.9)	123 (74.1)	
N classification						
N0	53 (55.2)	43 (44.8)	0.002	35 (36.5)	61 (63.5)	0.013
N1 + N2	28 (32.6)	58 (67.4)		17 (19.8)	69 (80.2)	
M classification						
M0	73 (48)	79 (52)	0.031	48 (31.6)	104 (68.4)	0.043
M1	8 (26.7)	22 (73.3)		4 (13.3)	26 (86.7)	

## Discussion

Chaperonin proteins are key molecular complexes that help proteins fold correctly, maintain energy stability and protein conformation, and are essential for cell survival and growth [24]. As an important subunit of the CCT complex, studies have shown that CCT8 is associated with the progression of colorectal cancer [25], breast cancer [26], lung cancer [27], and uterine sarcoma [28]. CCT8 is abnormally up-regulated in many kinds of tumors, which is related to the poor prognosis of tumor patients [29]. We used a variety of methods to detect the freshly matched tissues and paraffin samples of CRC, and found that CCT8 was highly expressed in CRC tissues, while low expression in normal CRC tissues. Kaplan–Meier plotter was analyzed by PrognoScan database to evaluate the correlation between the expression of CCT8 and the prognosis of patients with CRC. The results showed that the expression of CCT8 in patients with low overall survival rate was significantly higher than that in patients with high survival rate. Based on this, we speculate that CCT8 may be involved in the progression of CRC and play an important role in promoting CRC.

Proliferation, invasion, and metastasis are important signs of malignant progression of tumor cells. Studies have shown that CCT8 can promote the proliferation and invasion of hepatocellular carcinoma, esophageal squamous cell carcinoma, and glioma cells [7, 29–31]. CRC was classified into four subtypes with distinct molecular and biological characteristics: CMS1 (microsatellite instability immune), CMS2 (canonical), CMS3 (metabolic), and CMS4 (mesenchymal). Eight commonly used CRC cell lines were used in our experiment. According to previous reports, RKO and HCT116 belong to CMS1 [32]; Caco2 belongs to CMS4 [33, 34]; SW620 [32], HCT15 [32], HT29 [32], SW480 [34], and DLD1 [33, 34] are undefined subtype. However, since the most important purpose of our study is to observe the effect of CCT8 changes on the function of CRC cells, so we included all the available CRC cell lines in the experiment to obtain a more comprehensive CCT8 expression pattern. We chose RKO, SW620, SW480, and HCT116 cells to perform the in vitro experiments for their high transfection efficiency. We knocked down the expression of CCT8 in HCT116 and SW480 with relatively high expression of CCT8, and on the contrary to overexpressed the expression of CCT8 in RKO and SW620 with relatively low expression of CCT8. The results of in vitro functional experiments showed that CCT8 could significantly enhance the proliferation and invasion of CRC cells, while the results of subcutaneous tumorigenesis and cecal in situ implantation in nude mice showed that overexpression of CCT8 could promote the growth and metastasis of CRC cells. Thus, it can be seen that CCT8 is involved in the regulation of proliferation, invasion, and metastasis of CRC cells, and it is more important to clarify this regulatory mechanism.

As a molecule originally identified from breast axillary lymph node metastasis, the overexpression and nuclear localization of LASP1 are closely related to the malignant progression of breast cancer cells and the overall survival rate of patients with breast cancer [9]. Moreover, abnormal expression of LASP1 was detected in a variety of other cancers, including hepatocellular carcinoma [13], nasopharyngeal carcinoma [11], prostate cancer [14], pancreatic cancer [15], and ovarian cancer [12]. In CRC, LASP1 promotes the progression of CRC through multiple molecular and signal pathways [35]. In our previous study, several LASP1 regulatory proteins were screened by the proteomic strategy of two-dimensional differential gel electrophoresis (2-D DIGE). As one of them, the regulatory relationship between CCT8 and LASP1 is not clear. The interaction between CCT8 and LASP1 was verified by Co-IP test, and overexpression CCT8 recovery test was set up. Our results showed that LASP1 can bind to CCT8, and CCT8 can restore the ability of LASP1 to promote the invasion of colorectal cancer cells. This finding complements the molecular mechanism that LASP1 promotes the malignant progression of colorectal cancer, and further confirms that CCT8 plays a role in colorectal cancer through interaction with LASP1.

In order to further explore the possible mechanism of CCT8 in colorectal cancer, bioinformatics techniques were used to enrich and analyze the regulatory molecules or signal pathways downstream of CCT8. We found that p53 and its downstream cell cycle and EMT transformation signal pathways were significantly related to the expression of CCT8.

The tumor suppressor protein p53, known as “guardian of the Genome” [36], is a DNA-binding protein involved in apoptosis, cell cycle arrest, or DNA repair [37]. Deactivating p53 signaling either by altering p53 regulators or by p53 mutations occurs frequently in human colorectal carcinoma (CRC). A total of 43% of CRCs harbor p53 mutations that reduce WTp53 tumor suppressor activity and often contribute to tumorigenesis [38]. Meanwhile, many studies have observed the accumulation of p53 protein in malignant tumors, as well as during tumorigenesis, unrelated to p53 mutations [39, 40]. Immunocytochemistry have detected the immunolocalization of p53 protein in the cytoplasm, which may be a new mechanism of p53 inactivation, defined as nuclear rejection [41]. Previous studies have shown that there is an interaction between TCP1 and p53 in hepatocellular carcinoma [42]. We have confirmed that CCT8 is mainly expressed in the cytoplasm, so we speculate that CCT8 may play a tumor promoting role by interacting with p53 in the cytoplasm. In this study, the results of Co-IP, nuclear and cytoplasmic separation and immunofluorescence experiments confirmed that in p53 wild type colorectal cancer cell line, CCT8 interacted with p53 in the cytoplasm and inhibited p53 entry into the nucleus. The immunohistochemical results showed that the expression of CCT8 was positively correlated with the cytoplasmic expression of p53. However, interestingly, this phenomenon was not observed in p53 mutant colorectal cancer cell line HCT15, and the immunohistochemical results also showed that there was no significant correlation between the expression of CCT8 and MTp53. Those results indicate that CCT8 is closely related to the abnormal cytoplasmic localization of p53 by interacting with it, which maybe a new mechanism of p53 inactivation, but the interaction between CCT8 and p53 does not occur in the type of p53 mutated colorectal cancer.

It is well known that p53 is involved in cell cycle arrest and apoptosis. Our bio-informatics study found that there was a significant correlation between cell cycle signal pathway and the expression of CCT8. These results suggest that CCT8 may be involved in tumor cell cycle regulation related to cell proliferation and cancer progression. In this study, the results of nuclear cytoplasmic protein and cell cycle assay confirmed that in p53 wild type colorectal cancer cell line, CCT8 inhibited p53 entry into the nucleus, down-regulated cell cycle inhibitory proteins p21 and P27, and up-regulated cell cycle-related molecules (CDK4, CDK6, cyclinD1, myc), and promote tumor cell proliferation. In p53 mutant colorectal cancer cell line HCT15, CCT8 did not affect cell cycle progression.

It has been reported that p53 also plays a key role in EMT and metastasis [43]. P53 signal affects EMT transcription factors snail, slug, and twist. Most EMT transcription factors are transcriptional suppressors, including Snail, Slug, and twist. They inhibit epithelial-specific genes, especially molecules involved in stabilizing cell-cell connections, such as E-cadherin, and up-regulate components of the mesenchymal migration mechanism. p53 signal antagonizes EMT transcription factors, thus negatively regulating EMT [28, 44, 45]. Therefore, we speculate whether CCT8 may also regulate the EMT of CRC cells by inhibiting the entry of p53 into the nucleus? As expected, our results confirmed that CCT8 did antagonize the inhibition of EMT transcription factors, which in turn promoted the EMT progression of CRC cells. Similarly, this phenomenon was not observed in HCT15 cell line.

## Conclusion

In conclusion, this study shows that CCT8 can interact with LASP1 to promote the proliferation, invasion, and metastasis of CRC cells, and this effect is achieved by inhibiting the function of WTp53. Therefore, silencing the types of CRC with overexpression of CCT8 may be a promising method for the treatment of recurrence and metastasis of colorectal cancer.

## Supplementary information

Supplement material

## Data Availability

The datasets generated and/or analysed during the current study are not publicly available but are available from the corresponding author on reasonable request.
