# Supplementary material for: CCT8 recovers WTp53-suppressed cell cycle evolution and EMT to promote colorectal cancer progression
Source: Oncogenesis. 2021 Dec 3;10(12):84. doi: 10.1038/s41389-021-00374-3 (PMC8642402; doi:10.1038/s41389-021-00374-3)
Supplement: Supplementary file 1 — Supplement material [file 41389_2021_374_MOESM1_ESM.docx]

**Supplementary Information**

**CCT8 recovers WTp53-suppressed cell cycle and EMT to promote colorectal cancer progression**

**Supplementary Materials and Methods**

**Immunofluorescence (IF)**

Cells were cultured on confocal dishes overnight, fixed with 4% paraformaldehyde for 30 min and treated with 0.25% Triton X-100 for 10 min. After blocking in 10% normal blocking serum at room temperature for 30 min, dishes were incubated with rabbit anti-LASP1, rabbit anti-p53(1:200;Proteintech,Chicago,IL) and mouse anti-CCT8(1:100;Santa Cruz, California, USA) antibodies at 4^o^C overnight followed by washing with PBS three times. Dishes were then incubated with CoraLite594-conjugated antibodies and CoraLite488-conjugated antibodies (1:250; Proteintech, Chicago, IL) for 30min at room temperature, and then stained with cell plasma Membrane dye 3,3'-dioctadecyloxacarbocyanine perchlorate (DiO; Beyotime) and 6-diamidino-2-phenylindole (DAPI; Invitrogen).

**Proteomic analysis**

Conventional 2-D electrophoresis analysis and MS identification were performed as previously described [5]. For 2-D difference gel electrophoresis (2-D DIGE), the proteins were labelled with fluorescent cyanine dyes (GE Healthcare, Milwaukee, Wisconsin, USA) following the manufacturer’s instructions. In brief, 50mg of extracted protein to be compared was labelled with 400pmol Cy3 or Cy5, while 400pmol Cy2 was employed to label 50mg of internal standard protein of each sample at an equal amount. The labelling was performed on ice in the dark for 30min, and then quenched with 1ml of 10mM lysine (Sigma) for 10 min. A 50mg aliquot of Cy3- and Cy5-labelled samples was combined before mixing with 50mg of Cy2-labelled internal standard. The 2× sample buffer with an equal volume (8 M urea, 2 M thiourea, 4% CHAPS, 2% Bio-lyte, pH 4-7, 130mM dithiothreitol (DTT)) was added to the sample, and the final volume was adjusted to 450ml with rehydration buffer (8 M urea, 4% CHAPS, 1% Biolyte, pH 4e7, 40 mM DTT). The proteins were applied to IPG (immobilised pH gradient) strips (pH 4-7, 24cm) and focused on an IPGphor (GE Healthcare). Focused IPG strips were equilibrated, and then loaded onto 12% SDS-polyacrylamide gels (SDS-PAGE) using low-fluorescence glass plates on an Ettan DALT II system (GE Healthcare). All electrophoresis procedures were performed in the dark. The biological triplicates were run on three gels as analytical gels. In addition, another strip was performed in parallel as a preparative gel for picking spots as described in 2-D DIGE, except that the IPG strip was loaded with 1000mg of proteins, and the gel was stained with Coomassie brilliant blue. After SDS-PAGE, the three analytical gels were scanned with a Typhoon 9410 scanner (GE Healthcare) with appropriate excitation/emission wavelengths specific for Cy2 (488/520 nm), Cy3 (532/580 nm) and Cy5 (633/670 nm) to generate nine protein spot maps.

DeCyder 5.0 software (GE Healthcare) was used for 2-D DIGE analysis according to the manufacturer’s recommendation. The DeCyder differential in-gel analysis (DIA) module was used for pairwise comparisons of each sample with the internal standard in each gel. The DeCyder biological variation analysis (BV A) module was then used to simultaneously match all nine protein spot maps, using the Cy3/Cy2 and Cy5/Cy2 DIA ratios, to calculate average abundance changes and paired Student t test p values for the variance of these ratios for each protein pair across all samples. The differential protein spots (|ratio|>2, P<0.05) which were altered consistently in all three protein spot maps were selected for further identification.

**Cell proliferation assays**

Cell proliferation assays were carried out using Cell Counting Kit 8 (CCK8) (Dojindo; Kumamoto, Japan). Cells were plated in 96-well plates at a density of 1 × 10^4^ cells per well and cultured in the growth medium. The cells in the 96-well plate were sucked out of the primary culture and incubated with 100ul mixture (complete medium: CCK8 reagent = 9:1) at 37^o^C for 2-4 h. The OD at the 450nm of each hole was detected by enzyme-labeled instrument, and the blank control group was used as the benchmark to zero. The obtained OD value reflects the ability of cell proliferation. The average value of 5 multiple holes in each group was taken to draw the cell proliferation curve.

**Wound-healing and Transwell migration assays**

Wound-healing and Transwell migration assays were performed as previously described [3]. CRC cells were seeded onto 24-well plates and grown to confluence. After starvation in serum-free medium for 12 h, the cells were gently wounded using pipette tips. Images were taken at 0, 12 and 60 h after wounding. The motility of the CRC cells was assessed by the distance between the wound edges. Approximately 1.5-3×10^5^CRC cells cultured in 200μl of 1640 medium were seeded in the upper chamber of the Transwell insert, while 600μl of medium with 20% FBS was placed in the lower chamber. Cells were cultured for 24 h, and those invading the underside of the membrane were fixed and stained with crystal violet. Cells were counted from five random fields of view, and the mean value was calculated from three independent experiments.

**Cell cycle**

The Cell cycle was measured by cell cycle detection kit (Keygen; China). 1×10^6^ cells in good condition were collected, washed twice with cold PBS solution, digested with trypsin, centrifuged with 1000rpm for 5minutes, resuscitated in complete culture medium and counted. The number of cells collected is about 1×10^5^-1×10^6^. Wash the cells with PBS solution twice, each time 2000rpm centrifuge for 5minutes, discard PBS solution and drain on filter paper. 70% ethanol was added and fixed at 4^o^C for 2 hours, then centrifuged and washed with PBS solution twice, each time 2000rpm centrifuge for 5 minutes. Add 100ul RNase A and put it in water at 37 ^o^C for 30 min, then add 400ul PI to mix well, avoid light and incubate at 4 ^o^C for 30 min. After staining, it should be detected on the computer within 1 hour, and the red fluorescence at the excitation wavelength 488nm is recorded.

**Cell apoptosis**

The apoptosis of cell was measured by Annexin V-FITC/PI double staining apoptosis detection kit (Keygen; China).10×10^6^ cells in good condition were collected, washed twice with PBS solution, digested with trypsin without EDTA, re-suspended and counted after centrifugation. Collect about 1×10^5^-1×10^6^ cells. The cells were washed with PBS solution for 2 times and centrifuged with 2000rpm for 5 min each time. PBS solution was discarded and drained on filter paper. Binding-buffer 500ul resuspension cells were added to each tube, and then added 5ul Annexin V reagent to fully blow and mix. Finally, add 5ul Propidium Iodide test and the cells were incubated at room temperature and away from light for 5-15 minutes. After staining, the apoptosis rate of cells should be detected by flow cytometry within 1 hour.

**Co-immunoprecipitation (Co-IP)**

Cell extracts were incubated 2h at 4°C with IgG and protein A+G Agarose to get rid of non-specific binding. Primary antibodies were then added to separate cell extract tubes for incubation at 4°C overnight. The protein A/G-agarose was collected by centrifugation. Immunoprecipitated proteins were analyzed by SDS-PAGE (10%, Minigel) at 100 V for 1.5 hr. Membranes were blocked first before antibodies were added for incubation at 4°C overnight. The secondary antibodies were then incubated for 1h at room temperature before final detection with enhanced chemiluminescence (PerkinElmer Life Sciences).

**Bioinformatics analysis**

The gene mutation data for selected TCGA cancer cohorts were downloaded from CBioportal(<http://www.cbioportal.org/>). TCGAbiolinks [1] was used to download the gene expression data of colorectal adenocarcinoma. All correlation analyses of different data readouts were performed using the Spearman correlation method (cofunction via the *ggpubr* R package), differential genes expression analysis was performed via limma package [2].

Gene Set Enrichment Analysis (GSEA) is a computational method determining whether pre-defined sets of genes show statistically significant, concordant differences between two biological states. In our research, GSEA was utilized to identify the potential pathways associated with CCT8 expression.

Plots construction and statistical analyses were performed using R (Version 4.0.1, R Foundation, Vienna, Austria) with R packages including Cluster Profiler [4], DOSE, ggpubr and enrichplot. For statical analysis, the p-values were adjusted using the B & H adjustment method (stats R package) for the multiple comparisons, and two-sided P values <0.05 were considered to be statistically significant.

Gene expression data were available for 615 CRC patients. Expression levels were log2 transformed. Kaplan-Meier plotter analyzed by PrognoScan (http://dna00.bio.kyutech.ac.jp/PrognoScan/index.html) database was used to evaluate the prognostic relevance of CCT8 in univariate analysis.

**References**

1. Mounir M, Lucchetta M, Silva TC, Olsen C, Bontempi G, Chen X, et al. New functionalities in the TCGAbiolinks package for the study and integration of cancer data from GDC and GTEx. Plos Comput Biol. 2019;15**:** e1006701.

2. Ritchie ME, Phipson B, Wu D, Hu Y, Law CW, Shi W, et al. limma powers differential expression analyses for RNA-sequencing and microarray studies. Nucleic Acids Res. 2015;43**:** e47.

3. Yan P, Liu J, Zhou R, Lin C, Wu K, Yang S, et al. LASP1 interacts with N-WASP to activate the Arp2/3 complex and facilitate colorectal cancer metastasis by increasing tumour budding and worsening the pattern of invasion. Oncogene. 2020;39**:** 5743-5755.

4. Yu G, Wang LG, Han Y, He QY. clusterProfiler: an R package for comparing biological themes among gene clusters. OMICS. 2012;16**:** 284-287.

5. Zhao L, Wang H, Li J, Liu Y, Ding Y. Overexpression of Rho GDP-dissociation inhibitor alpha is associated with tumor progression and poor prognosis of colorectal cancer. J Proteome Res. 2008;7**:** 3994-4003.

**Supplementary Figures**

**Figure S1, related to Figure 1. LASP1 positively regulates CCT8 expression by protein interactions.** Representative MS/MS spectrum of the doubly charged precursorion obtained from 2-D difference gel electrophoresis after trysin digestion.


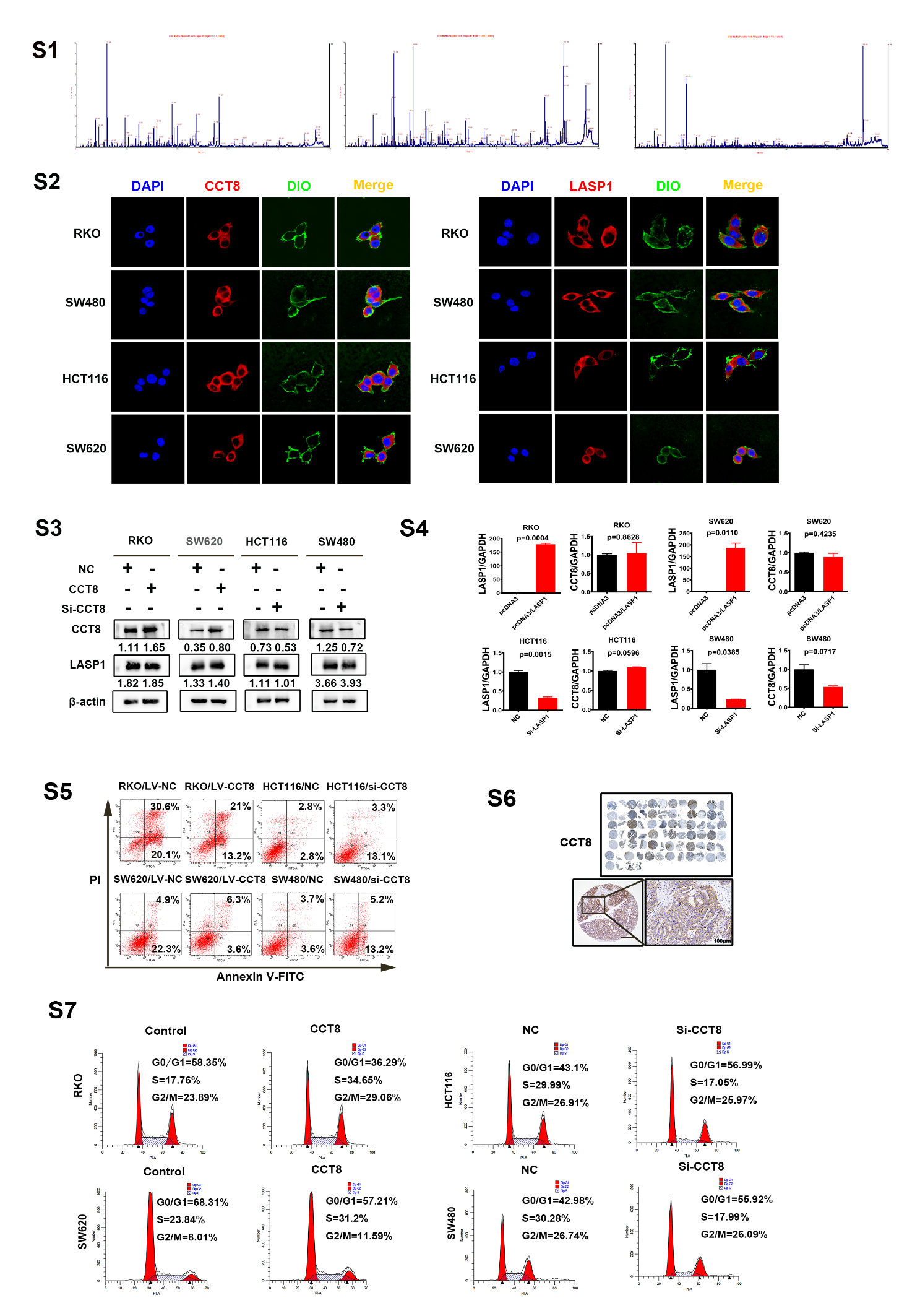


**Figure S2, related to Figure 1. LASP1 positively regulates CCT8 expression by protein interactions.** The immunofluorescence staining was performed to assess the subcellular localization of CCT8 and LASP1 in indicated cells (original magnification ×2400). Dio dye was used in cell membrane labeling The box areas highlighted the co-localization between CCT8 and LASP1.


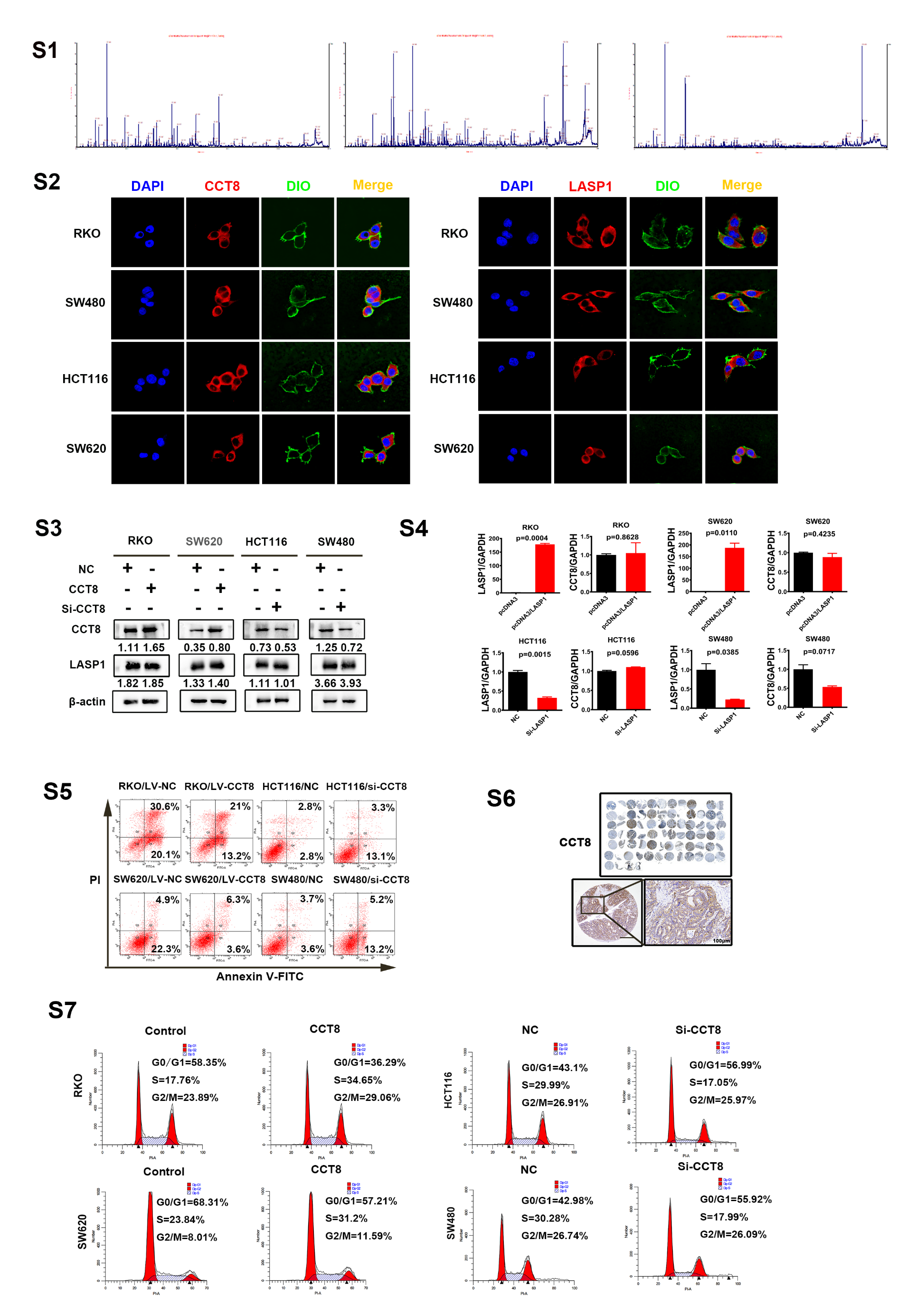


**Figure S3, related to Figure 1. LASP1 positively regulates CCT8 expression by protein interactions.** Western Blot was performed to detect the expression of CCT8 and LASP1 protein in indicated cells.


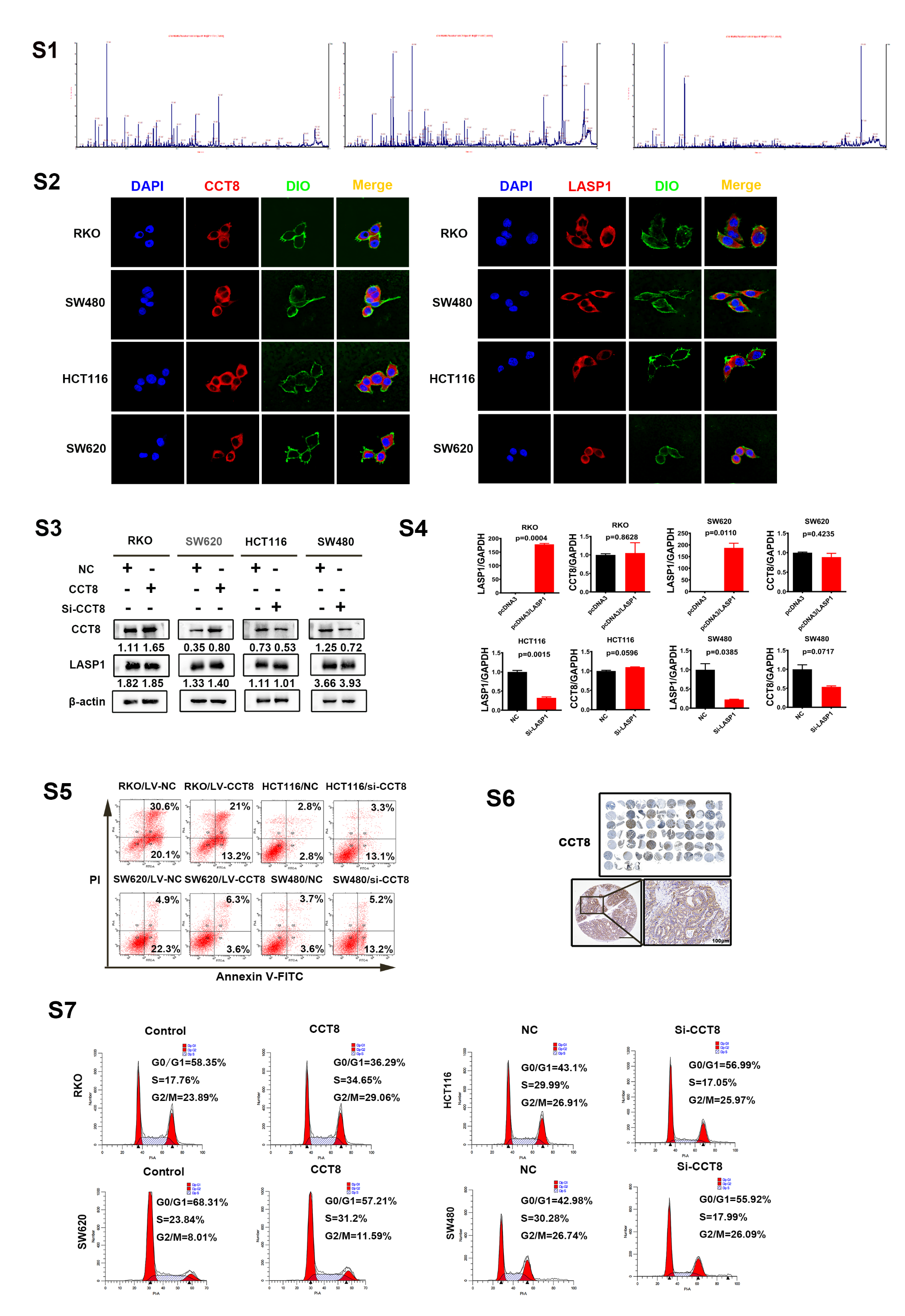


**Figure S4, related to Figure 1. LASP1 positively regulates CCT8 expression by protein interactions.** RT-PCR assay was performed to detect the expression of CCT8 and LASP1 mRNA in indicated cells.


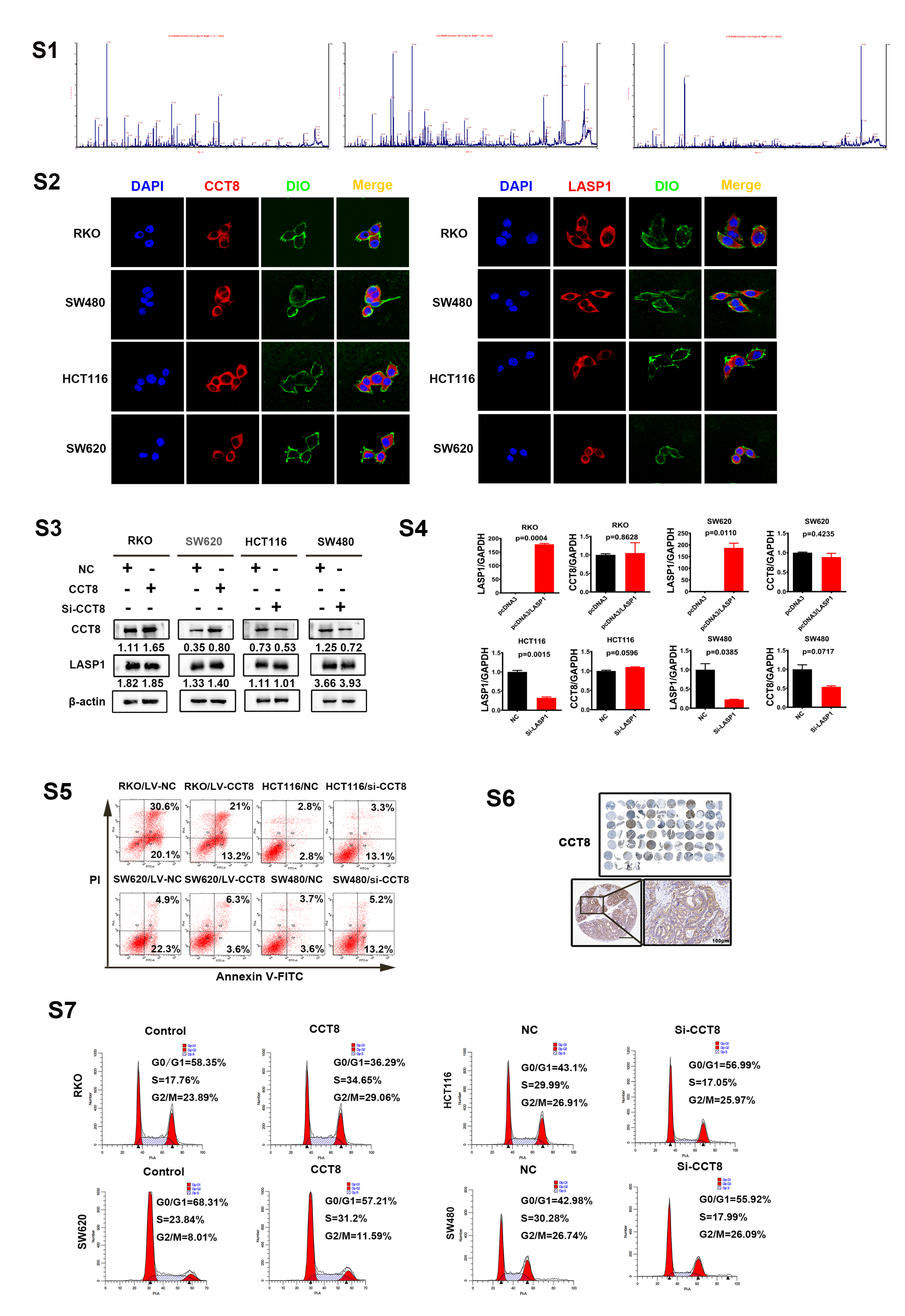


**Figure S5, related to Figure 3. CCT8 promoted the proliferation of CRC cells in vivo and in vitro.** Flow cytometric analysis of apoptosis in vitro after treatment of fluorouracil for 12h.

**
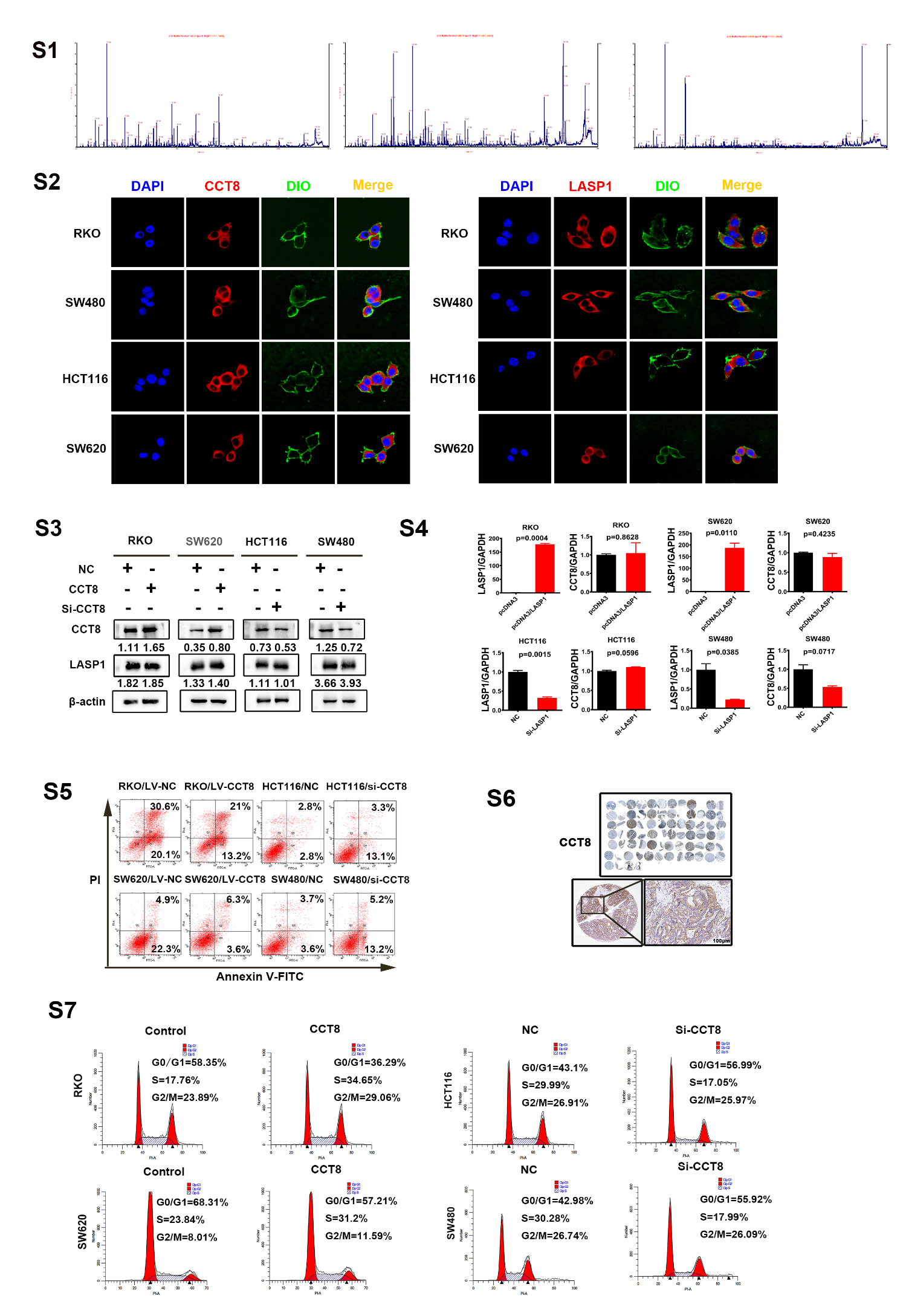
**

**Figure S6, related to Figure 3. CCT8 promoted the proliferation of CRC cells in vivo and in vitro.** Representative figures and data of cell cycle analysis in CCT8 overexpressed and silenced CRC cell lines.

**
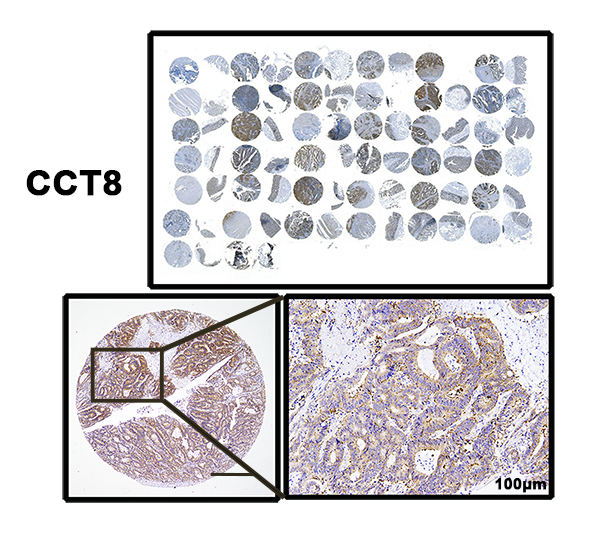
**

**Figure S7, related to Figure 6. The high expression of CCT8 is associated with the progression and poor prognosis of colorectal cancer.** The expression CCT8 was detected in tissue chips using IHC staining.

**Supplementary Table S1. siRNA sequences used for transfection**

| **Gene name** | **Sense 5’-3’** | **Antisense 5’-3’** |
| --- | --- | --- |
| **CCT8-si-1531** | GCUGAGGCAUUUGAAGCUATT | UAGCUUCAAAUGCCUCAGCTT |
| **CCT8-si-1683**  **CCT8-si-1901** | GCUGGAAGCUGGUAUUCUATT  ACUCAAGAAUCACCUGAUGTT | UAGAAUACCAGCUUCCAGCTT  CAUCAGGUGAUUCUUGAGUTT |
| **LASP1-siRNA** | GGUGAACUGUCUGGAUAAGTT | CUUAUCCAGACAGUUCACCTT |
| **Negative control (NC)** | UUCUCCGAACGUGUCACGUTT | ACGUGACACGUUCGGAGAATT |

**Supplementary Table S2. RT-PCR primer sequences for human genes**

| **Gene name** | **Forward primer 5’-3’** | **Reverse primer 5’-3’** |
| --- | --- | --- |
| **CCT8** | AGGACATGCTGGAAGCTGGT | CTTCCCACTTGGAGGCTTGG |
| **LASP1** | CGAGAAGAAGCCCTACTGCAA | CTGCCACTACGCTGAAACCT |
| **GAPDH** | CTCCTCCTGTTCGACAGTCA | TGCAGGAGGCATTGCTGATG |
